# Javanese Turmeric (*Curcuma xanthorrhiza* Roxb.): Ethnobotany, Phytochemistry, Biotechnology, and Pharmacological Activities

**DOI:** 10.1155/2021/9960813

**Published:** 2021-06-11

**Authors:** Endang Rahmat, Jun Lee, Youngmin Kang

**Affiliations:** ^1^University of Science & Technology (UST), KIOM Campus, Korean Convergence Medicine Major, Daejeon 34054, Republic of Korea; ^2^Herbal Medicine Resources Research Center, Korea Institute of Oriental Medicine, 111 Geonjae-ro, Naju-si, Jeollanam-do 58245, Republic of Korea

## Abstract

*Curcuma xanthorrhiza* Roxb., locally famed as Temulawak, has been extensively utilized in Indonesia as medicinal and nutritional plants since immemorial time. The rhizome of this plant is an important ingredient for *jamu* formulation (Indonesian traditional medicine). *C. xanthorrhiza* is traditionally used to treat several ailments such as lack of appetite, stomach disorder, liver illness, constipation, bloody diarrhea, dysentery, arthritis, children's fevers, hypotriglyceridaemia, hemorrhoids, vaginal discharge, rheumatism, and skin eruptions. To date, over 40 active compounds, including terpenoids, curcuminoids, and other phenolic compounds, have been isolated and identified from *C. xanthorrhiza* Roxb. Some pharmacological tests reported that *C. xanthorrhiza* Roxb. has antioxidant, antimicrobial, anti-inflammatory, anticancer and antitumor, antidiabetic, and skincare and hepatoprotective properties. Efforts for biotechnologically production of *C. xanthorrhiza* have also been conducted, resulting in some micropropagation protocols of this plant. The current review focuses on the botanical description and distribution, ethnomedicinal uses, production and conservation status, phytochemical properties, and pharmacological activities of *C. xanthorrhiza* Roxb. to provide accurate and reliable data for future researches and commercialization purposes.

## 1. Introduction

*Curcuma xanthorrhiza* Roxb. (family: Zingiberaceae) is a native Indonesian highly valuable plant locally familiar as “Temulawak” or Java turmeric. It is mainly cultivated in Indonesia and other Southeast Asian countries such as Malaysia, Thailand, Vietnam, and Phillippines [1, 2]. Recorded in 2019, *C. xanthorrhiza* was cultivated in Indonesia on a massive scale with a demolished harvested area of more than 13,042,873 m^2^ producing yields of 29,637,119 kg [3].

Traditionally, *C. xanthorrhiza* have been greatly harnessed all over its local distribution area as an ingredient of *jamu* (Indonesian herbal supplement and medicine) or to medicate and control numerous of sickness and disorders since ancient time including lack of appetite, stomach illness, liver ailments, constipation, bloody diarrhea, dysentery, arthritis, children's fevers, hypotriglyceridemic, hemorrhoids, vaginal discharge, rheumatism, and skin eruptions [4–9]. The efficacy of *C. xanthorrhiza* for treating various diseases has been confirmed due to having pharmacology properties such as anti-inflammatory, antibacterial, antioxidative, neuroprotective, nephroprotective, antitumor, and hepatoprotective activities [10–18]. *C. xanthorrhiza* (Temulawak) has also been exported and utilized in Europe at least since 1963, particularly for the treatment of dyspepsia, infections, and skin and liver illness.

Scientific studies showed that the most abundant essential phytochemicals obtained from the *C. xanthorrhiza* rhizome were terpenoids and curcuminoids [19–21]. Therefore, the medicinal activity of *C. xanthorrhiza* is mostly caused by these two major groups of compounds [22]. The main medicinal part of *C. xanthorrhiza* which contains many sesquiterpenoids and curcuminoids is rhizome [19]. Hence, market demand for rhizome of *C. xanthorrhiza* is increasing from time to time.

To answer the challenge of increasing global market demand for *C. xanthorrhiza*, several biotechnology studies through in vitro propagation and production have been carried out. Biotechnological treatments such as elicitation with yeast extract and the addition of colchicine have been carried out to enhance the growth of biomass and accumulation of active compound content of *C. xanthorrhiza* [23, 24]. Even so, biotechnology research for this plant is still very limited, and further comprehensive research is needed.

However, although there are many reports that explain the identification of secondary metabolites and pharmacological activities of *C. xanthorrhiza*, there is no comprehensive review published for critically summarizing these studies and offering potential future applications for both research and treatment. Therefore, here, we review this valuable medicinal plant in terms of botany and taxonomy, geographical distribution, traditional medicinal uses, active compounds, and pharmacological properties.

## 2. Materials and Methods

In this review, we deeply searched all the relevant works of literature related to botany, traditional/medicinal application, phytochemicals, safety evaluation, and pharmacological attributes of *C. xanthorrhiza.* To collect all published data about this species, we used international and local scientific databases including but not limited to PubMed, Scopus, Google Scholar, Web of Science, SINTA, ScienceDirect, and Wiley Online for all accessible papers. Several key terms were used: (“*Curcuma xanthorrhiza*” OR “Temulawak” OR “Javanese Turmeric”) AND (“Botany” OR “Medicinal application” OR “Traditional uses” OR “Toxicity” OR “Pharmacological properties” OR “Biological attributes” OR “Active compounds” OR “Diseases treated” OR “Herbal medicinal use”). Published scientific reports on *C. xanthorrhiza* in different languages (Bahasa, Malay, and English) were cited in this study. Secondary data were collected and examined, and conclusions were made appropriately.

## 3. Botanical Description

**Figure 1:**
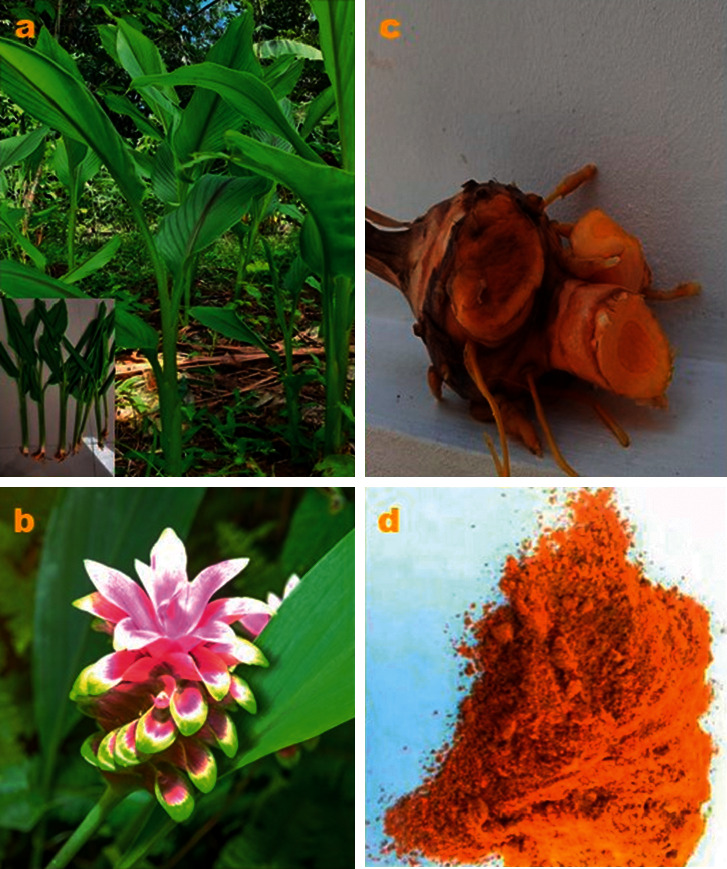
*Curcuma xanthorrhiza* Roxb. (a) Aerial parts of the plant. (b) Flower. (c) Rhizome. (d) Rhizome powder.

*C. xanthorrhiza* is an annual plant that grows in clumps and has a pseudostem (2–2.5 m in height) (Figure 1). Each colony consists of several plants (tillers as much 3 to 9 plants), and each plant has 2–9 leaves. *C. xanthorrhiza* leaves are long and quite wide (50–55 cm long leaves with a width of about 18 cm) [25]. *C. xanthorrhiza* flowers continuously throughout the year alternately coming out of the rhizome. The length of the flower stalk is about 3 cm, and the flower arrangement (*inflorescentia*) reaches 1.5 cm. One armpit consists of 3–4 flowers. Flower stalks are slim and hairy with a length of 4–37 cm. The grain is flower-shaped, elongated round whose length can reach 23 cm. *C. xanthorrhiza* flowers have many protective leaves whose length exceeds or sometimes is proportional to the length of the flower crown. Flowers bloom in the morning and wither in the afternoon [26]. The shape of the parent rhizome of *C. xanthorrhiza* is oval round like an egg, while the shape of branch rhizome found on the side part is elongated. Each plant has approximately 3–4 branch rhizomes. *C. xanthorrhiza* has a fibrous root system with a root length of about 2.5 cm with irregular location [25].

## 4. Taxonomy and Geographic Distribution

**Figure 2:**
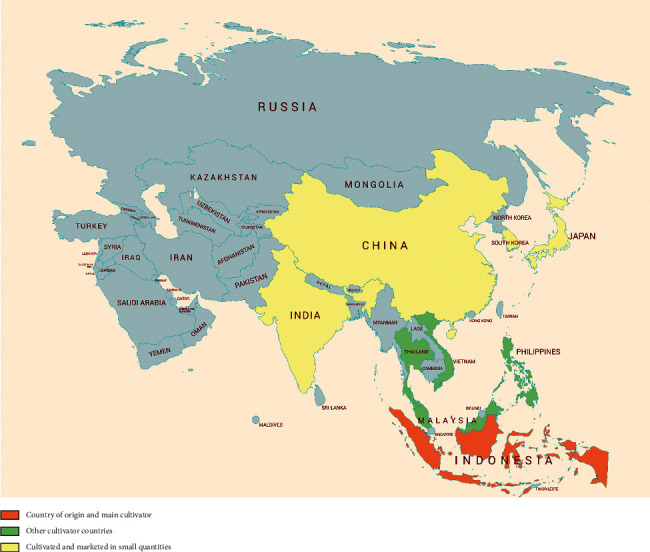
Geographic distribution of *Curcuma xanthorrhiza* Roxb.

*C. xanthorrhiza* Roxb. is an Indonesian original plant which belongs to the Zingiberaceae family and is found in many tropical regions (Figure 2). Besides being known as Temulawak or Javanese turmeric, *C. xanthorrhiza* has several regional names such as koneng gede (Sundanese), temu labak (Madurese), tommo (Bali), tommon (South Sulawesi), and karbanga (Ternate). This plant can grow in the lowlands to an altitude of 2500 m above sea level [27]. In Indonesia, this plant is widespread and cultivated in almost all major islands such as Java, Sumatra, Kalimantan, Sulawesi, and Maluku. In addition, *C. xanthorrhiza* has also been cultivated in several Southeast Asian countries such as Malaysia, Thailand, the Philippines, and Vietnam [1, 2]. In addition, cultivars may also be found in China, India, Japan, and Korea.

## 5. Ethnomedicinal Uses

In Indonesia, *C. xanthorrhiza* is a very well-known herb and has been widely applied by various tribes to treat various diseases (Table 1). Some traditional uses of *C. xanthorrhiza* are to overcome lack of appetite, constipation, hemorrhoid, acne, diarrhea, and seizure medication, to destroy gallstones, to treat kidney and liver diseases, rheumatic pain, rheumatism, and arthritis, and to treat thrush and vaginal discharge [39]. *C. xanthorrhiza* (Temulawak) has also been exported and utilized in Europe at least since 1963, particularly for the treatment of dyspepsia, infections, and skin and liver illness. Traditionally, *C. xanthorrhiza* rhizome is usually consumed in the form of fresh, decoction, steeping, powder, and even as food. Industrially, the semifinished product from the rhizome of *C. xanthorrhiza* is simplicia, starch, essential oil, and extract, while the final industrial products are food and drink, cosmetics, syrup, instant powder, tablets, and capsule. *C. xanthorrhiza* is also often combined with other medicinal plants into a mixture of jamu (Indonesian herbal medicine) products that have been widely produced commercially in Indonesia. For example, a mixture of *C. xanthorrhiza*, *Curcuma longa* Linn. syn., and *Zingiber officinale* is believed by the Javanese to increase stamina, maintain the immune system, and maintain the health of the digestive system. Furthermore, *C. xanthorrhiza* with *Tinospora cordifolia* and *Andrographis paniculata* is widely used by the people of Sulawesi as a cure for gastric disease [33].

However, our evaluation found that most of those traditional uses have been scientifically examined. Some of the scientifically proven ethnomedical uses of *C. xanthorrhiza* including the medication of skin disease, acne, and gastrointestinal problems such as dysentery, diarrhea, hemorrhoid, constipation, and other digestive system problems are related to its antimicrobial activity (Table 2). The efficacy of *C. xanthorrhiza* to treat arthritis is probably related to its anti-inflammatory ability [14, 52]. *C. xanthorrhiza* usage to treat some disorders related to metabolic syndrome such as hypotriglyceridemia has also been scientifically examined [53, 54]. The use of *C. xanthorrhiza* to cure liver illness has also been tested scientifically [15, 18, 55, 56]. We believe that the traditional efficacy of *C. xanthorrhiza* is mostly related to its antimicrobial, antioxidant, and anti-inflammatory capability. Moreover, the efficacy of *C. xanthorrhiza* for malaria treatment has been evaluated preliminarily by heme polymerization inhibition [57]. Surely, this antimalaria activity of *C. xanthorrhiza* has to be evaluated more comprehensively. On the other hand, we have not found trusted scientific proof indicating *C. xanthorrhiza* effectiveness for the treatment of gynecology-related disorders such as vaginal discharge, postpartum medicine, and postdelivery concoctions. These traditional uses have to be evaluated more using a scientific approach.

## 6. Phytochemical Properties of *C. xanthorrhiza* Roxb

**Figure 3:**
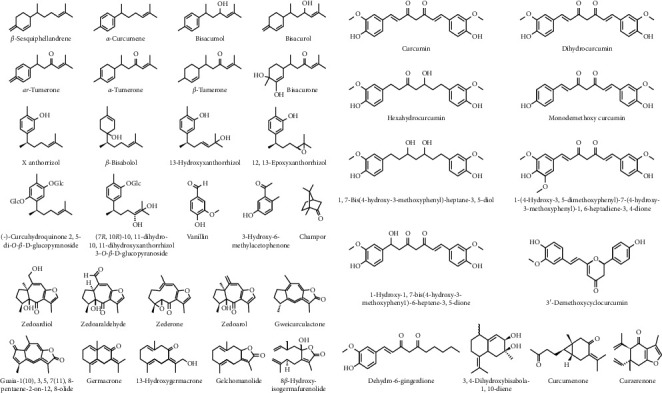
Isolated active compounds from *Curcuma xanthorrhiza* Roxb.

Chemical studies of *C. xanthorrhiza* established a foundation of pharmacological research. Substantial works have been done to isolate and purify active compounds from this valuable herb, resulting in the identification of several secondary metabolites (Figure 3). Among these chemicals, curcuminoids and terpenoids are the majority, and they own essential biological properties [19–21]. Furthermore, based on various studies, apparently, the majority in the quantity of the secondary metabolites obtained from the essential oil of *C. xanthorrhiza* rhizome is xanthorrhizol [58]. The presence of xanthorrhizol in *C. xanthorrhiza* is species-dependent, distinguishing *C. xanthorrhiza* (Temulawak) from *C. longa* (turmeric) [19, 59].

However, there is a difference in metabolite profiles of *C. xanthorrhiza* rhizome in diverse fractions (methanol, ethyl acetate, chloroform, and hexane) of ethanol extract. Terpenoids such as xanthorrhizol were prominent in the hexane soluble fraction. Large amounts of phenolic compounds, such as curcuminoids, were present in ethyl acetate soluble fractions. Amino acids, including methionine, phenylalanine, tryptophan, valine, alanine, lysine, and leucine, were discovered in the methanol fraction. The hexane, chloroform, and ethyl acetate fractions did not have carbohydrate and amino acid signals [60]. This is because the difference in the polarity of the solvent results in the discrepancy in the retention of different types of plant compounds. Below are the major group of secondary metabolites present in the rhizome of *C. xanthorrhiza*.

### 6.1. Terpenoids

Various studies have shown that *C. xanthorrhiza* contains a variety of terpenoids, especially bisabolane sesquiterpenoids. Earlier, at least 50 bisabolane sesquiterpenes were discovered from the genus *Curcuma*, particularly its turmeric rhizome [61]. Sesquiterpene compounds that have been successfully isolated and characterized from *C. xanthorrhiza* are bisacurone, bisacumol, bisacurol, curlone [62], *α*-curcumene, *ar*-turmerone, xanthorrhizol, germacrone, *β*-curcumene, *β*-sesquiphellandrene, curzerenone, *α*-turmerone, *β*-turmerone [62], (7*R*,10*R*)-10,11-dihydro-10,11-dihydroxy-xanthorrhizol 3-*O*-*β*-D-glucopyranoside, (-)-curcuhydroquinone 2,5-di-*O*-*β*-D-glucopyranoside, 13-hydroxy-xanthorrhizol, 12,13-epoxy-xanthorrhizol, *β*-bisabolol [61], zedoardiol, 3,4-dihydroxybisabola-1,10-diene, zedoarol, guaia-1(10),3,5,7(11),8-pentaene-2-on-12,8-olide, zederone, curcumenone [21], zedoaraldehyde, gweicurculactone, 13-hydroxygermacrone, germacrone, gelchomanolide, and 8*β*-hydroxy-isogermafurenolide [63]. In addition to sesquiterpenes, *C. xanthorrhiza* also contains monoterpene which has been successfully isolated, namely, camphor [19].

### 6.2. Curcuminoids

Curcuminoids are natural polyphenol compounds with a linear diarylheptanoid structure. A curcuminoid of *C. xanthorrhiza* mostly consists of dicinnamoylmethane derivatives. Various curcuminoids have been successfully obtained and categorized from the rhizome of *C. xanthorrhiza* such as octahydrocurcumin, 1-hydroxy-1,7-bis(4-hydroxy-3-methoxypheny1)-6-heptene-3,5-dione, dihydrocurcumin, hexahydrocurcumin, curcumin [62], monodemethoxy curcumin, bis-demethoxy curcumin [19], 1-(4-hydroxy-3,5-dimethoxyphenyl)-7-(4-hydroxy-3-methoxyphenyl)-1,6-heptadiene-3,4-dione [13], 3-demethoxycyclocurcumin, and 1,7-bis(4-hydroxy-3-methoxyphenyl)-heptane-3,5-diol [60].

### 6.3. Other Phenolic Compounds

In addition to terpenoids and curcuminoids, several other chemical compounds have also been successfully detected in the rhizome of *C. xanthorrhiza* such as vanillin [21], 3-hydroxy-6-methylacetophenone, and dehydro-6-gingerdione [63]. However, 13-hydroxy-xanthorrhizol and 12,13-epoxy-xanthorrhizol were identified beforehand from *Curcuma longa* rhizomes. Meanwhile, 3-hydroxy-6-methylacetophenone alluded as a residual product of synthetic reactions [64, 65], but Zhang et al. [63] found it for the first time as a nature-derived metabolite. Moreover, Zhang et al. [63] also announced the first identification of dehydro-6-gingerdione from *C. xanthorrhiza*, which was previously isolated from *Zingiber officinale* [66].

Mostly, the active compounds above were isolated from the rhizome of *C. xanthorrhiza*. However, other parts of *C. xanthorrhiza* are also probably containing a variety of important secondary metabolites, for example, the flower bract, which is proven to contain several active compounds, namely, xanthorrhizol (16.13%), *α*-curcumene (15.12%), *β*-elements (4.60%), trans-caryophyllene (3.48%), *β*-farnesene (0.29%), camphor (0.21%), and isoborneol (0.04%) [47].

## 7. Biotechnological Production of *C. xanthorrhiza* Roxb

High market demand for *C. xanthorrhiza* as one of the important sources of herbal medicine ingredients both in Indonesia and in other countries triggers the implementation of several studies on the production technology of *C. xanthorrhiza* plants which are effective, efficient, and safe and of high quality. One of them is micropropagation through plant tissue culture techniques. Several combinations of growth media and hormones have been used as a protocol for micropropagation of *C. xanthorrhiza* seedlings including a combination of MS media + IBA + BAP [67], a combination of MS media + BA + NAA [68], and a combination of MS media + BAP for microrhizome formation [69]. The above studies only use MS medium as their propagation protocol. Therefore, to further elaborate the study in order to find the most optimum growth condition for *C. xanthorrhiza*, it is necessary to try another type of growth media such as woody plant medium (WPM), Linsmaier and Skoog (LS) medium, Gamborg (B_5_) medium, and Nitsch and Nitsch (NN) medium. In addition, to obtain high-quality clone results, it is necessary to test the genetic fidelity, chemical property, and physiological characteristics between the mother plant and the clone. Another strategy that has been carried out to increase the average number of shoots and roots of *C. xanthorrhiza* is by inducing polyploidy which can be achieved by colchicine feeding [23]. Natural materials that are easily found such as coconut water are also proven to stimulate the growth of in vitro seedling of *C. xanthorrhiza* and contain more chlorophyll compared to *C. xanthorrhiza* that grows conventionally [70]. In addition, efforts have also been made to increase the bioactive compound content within *C. xanthorrhiza*. One of the ways is by using elicitation technique by yeast extract which is proven to increase the content of xanthorrhizol [23]. However, all of the above studies are still preliminary, and there is still plenty of research space that can be explored for optimum propagation and production of *C. xanthorrhiza* including the enhancement of its phytochemicals content by well-known elicitors such as methyl jasmonate and salicylic acid, as well as its scale-up production system using bioreactor.

## 8. Pharmacological Activities of *C. xanthorrhiza*

Different biological activities of *C. xanthorrhiza* have been studied using various approaches. Many of the examined pharmacological attributes bolster the unconfirmed ethnomedicinal application of this herb. Pharmacological reports in detail are explained hereinafter.

### 8.1. Antioxidant Activity

Aerobic metabolism in the human body could produce free radicals (e.g., nitric oxide, superoxide, and hydroxyl radicals) and other reactive species (e.g., peroxynitrite, hydrogen peroxide, and hypochlorous acid) [71]. Free radical reactions can cause harmful degenerative illness including degenerative eye disorder, senile dementia, asthma, diabetes, atherosclerosis, cancer, and inflammatory joint sickness [72]. Antioxidants are compounds that exert synergistic actions in scavenging free radicals by inhibition of oxidation reaction. Some studies reported the potential natural antioxidant activity of *C. xanthorrhiza* extract [73–75]. The antioxidant ability of *C. xanthorrhiza* has been evaluated using various methods including the DPPH (1,1-diphenyl-2-picrihydazi), superoxide anion, ferric reducing antioxidant power (FRAP), and metal bonding activity [76, 77].

Rosidi et al. [74] assessed the antioxidant activity of ethanol extract of *C. xanthorrhiza* in the liquid-liquid extraction method in hexane solvent using the DPPH method. The study revealed that the tested extract of *C. xanthorrhiza* has a relatively active antioxidant activity with an IC_50_ value of 87.01 ppm. Meanwhile, Widyastuti et al. [75] examined the antioxidant properties of *C. xanthorrhiza* harvested from different locations. The methanol extract of *C. xanthorrhiza* from various places has been assessed for its antioxidant properties along with total phenolic and total flavonoid composition. The result showed that *C. xanthorrhiza* has potent antioxidant activity and its level is apparently affected by different harvesting areas [75]. The discovery validates the diverse uses and effectiveness of this herb over various traditional areas and populations (Table 1) and gives possible suggestions on the levels of active molecules depending on the location of cultivation. Storage time following the harvesting also affects the antioxidant level of *C. xanthorrhiza*. The prolonged storage time of *C. xanthorrhiza* could decrease its antioxidant ability [78]. In addition, the harvesting age of *C. xanthorrhiza* is another parameter affecting the level of its antioxidant activity. A study by Rosiyani [79] showed that 9-month-old *C. xanthorrhiza* rhizome possesses greater antioxidant activity compared to 7- or 8-month-old harvesting time. This could happen probably because the curcuminoid content is higher in that of older *C. xanthorrhiza.* Along with *Curcuma longa* and *Zingiber officinale*, the antioxidant activity of methanol extract of *C. xanthorrhiza* was shown higher than the other seven Zingiberaceae species studied by Akinola et al. [80]. In addition, the antioxidant activity of pure xanthorrhizol from *C. xanthorrhiza* has been evaluated in copper-mediated isolated human low-density lipoprotein (LDL) oxidation [81] and sturdily reduced human LDL peroxidation in a dose-dependent manner. We recommend that xanthorrhizol could be tested for future experiments in cardiovascular disorders because strong LDL antioxidant properties could lower the risk of heart disorders. Future study of antioxidant test should also be performed in vivo.

We believe that the antioxidant properties of *C. xanthorrhiza* greatly contribute to its traditional uses. For example, traditionally, *C. xanthorrhiza* has been utilized as an arthritis medication where one of its major causes is the inflammation reaction within the joint caused by the free radical reaction. The antioxidant ability of *C. xanthorrhiza* in scavenging free radicals could treat this inflammatory joint sickness as reported traditionally. Furthermore, the traditional efficacy of *C. xanthorrhiza* for skin treatment is also inseparable from this antioxidant ability. The antioxidant activity has been reported to have connected with the prevention of skin aging [82].

However, although the potent antioxidant capability of *C. xanthorrhiza* was evaluated, it still leaves the fundamental question of whether all free radical scavenging activities are a reflection of the extracts and chemicals being tested and should be further evaluated using a more reliable antioxidant assay method. One of them is by using a more comprehensive in vitro cell-based antioxidant assay approach because it can detect metabolites that play a direct role in the antioxidant pathway and safeguarding cells against oxidative harm [83].

### 8.2. Antibacterial, Antifungal, and Anti-Insecticidal Activities

The antimicrobial activity of *C. xanthorrhiza* has been well studied using a number of disease-causing microbes (Table 2). The tested pathogens could cause some serious diseases such as food-borne illness, dental plaque, skin disease, infectious disease, pneumonia, tuberculosis, pulp necrosis, diarrhea, typhus, acne, nosocomial infections, hospital-acquired infection, filamentous fungal infections, nail infection, and penicilliosis (Table 2). Some well-known methods used to evaluate in vitro susceptibility test of *C. xanthorrhiza* such as NCCLS (M38-A) standard method and biofilm quantification, minimum bactericidal concentration (MBC), minimum inhibitory concentration (MIC), and minimum fungicidal concentration (MFC). Rhizome extract of *C. xanthorrhiza*, pure xanthorrhizol, and its essential oils are proven to be the potential of inhibiting or killing pathogenic microbes ranging from medium level activity to strong killing ability (Table 2) [40–42, 44–46, 48, 50, 84].

The antimicrobial capability of *C. xanthorrhiza* is possibly due to its phenolic compound content particularly xanthorrhizol and curcuminoids as the main ingredient. The phenolic compound has been reported to have inhibition action on cell walls or membranes of microbes by altering its cell permeability, resulting in the forfeit of essential molecules such as ATP, RNA, protein, and DNA [85]. Although the mode of antimicrobial action of xanthorrhizol has not been comprehensively elucidated, xanthorrhizol possibly has suppression ability on nuclear factor kappa B (NF-kB) and mitogen-activated protein kinase (MAPK) caused by microbial attack [86]. As for curcumin, some of its antimicrobial modes of action were explained by restraint of cytokinesis and bacterial cell multiplication, as well as interruption of bacterial cell wall and membrane, inducing cell lysis [87]. Curcumin was also reported to form electrostatic and/or hydrophobic interplay with cell membrane and wall of fungi resulting in a disarrangement of the membrane [88]. Even though the antibacterial attribute of *C. xanthorrhiza* seems to work on Gram-positive and some Gram-negative bacteria, it is more powerful than Gram-positive bacteria due to the composition of its cell wall.

Most of those reported microbe-related illnesses are linked up to the traditional uses of *C. xanthorrhiza* such as treatment of gastrointestinal problems and skin-related disorders. Thus, the reported antimicrobial activity of *C. xanthorrhiza* supports some of its traditional medicinal benefits. For example, the ability of ethanol extract of *C. xanthorrhiza* to suppress the growth of *Escherichia coli* is related to its traditional use for diarrhea treatment. In addition, the capability of its essential oil of flower bract to inhibit *Propionibacterium acnes* growth is probably related to its traditional usage for the medication acne. However, even if the antibacterial ability of this herb has been explained, their utilization as alternatives to antibiotics still needs pharmacodynamic and pharmacokinetic examination to reveal the mode of action of underlying metabolites.

Furthermore, pure xanthorrhizol isolated from *C. xanthorrhiza* extract has also reported anti-insecticidal properties against *Spodoptera littoralis* following its topical application [89]. This discovery opens its future potential for treating agriculture pests.

### 8.3. Anti-Inflammatory Property

The anti-inflammatory capability of methanol rhizome extract of *C. xanthorrhiza* has long been investigated on carrageenan-induced edema, vascular permeability caused by acetic acid, and the writhing phenomenon in rats [10]. The extract exhibited anti-inflammatory potential particularly by the presence of germacrone. The anti-inflammatory ability of *C. xanthorrhiza* probably is also related to its curcuminoid content especially curcumin. Curcumin has more potent anti-inflammatory properties than other curcuminoid derivatives such as demethoxy- or bisdesmethoxy-form [89].

Furthermore, the anti-inflammatory ability of *C. xanthorrhiza* is indeed also caused by xanthorrhizol as its marker compound. The earliest in vitro anti-inflammatory activity of xanthorrhizol has been reported on lipopolysaccharide-induced mice leukemic monocyte-macrophage cell RAW 264.7 [52]. The study reported the inhibition of prostaglandin E2 (PGE2) and nitric oxide (NO) production by xanthorrhizol, resulting in the reduction of cyclooxygenase-2 (COX-2) and inducible nitric oxide synthase (iNOS), respectively [52]. The result is supported by other anti-inflammatory tests of xanthorrhizol using lipopolysaccharide-activated primary cultured microglial cells [14]. Lim et al. [14] successfully showed that xanthorrhizol could hinder COX-2, iNOS, tumor necrosis factor-*α* (TNF-*α*), and proinflammatory cytokine interleukin-6 (IL-6) in induced microglial cells. Xanthorrhizol has also been studied in vivo using 12-*O*-tetradecanoylphorbol-13-acetate- (TPA-) activated mice severe inflammation specimen and restrains the impact of TPA-activated ornithine decarboxylase (ODC), COX-2, and iNOS expression in mice dermis [90]. According to these findings, xanthorrhizol can be confirmed to be involved in the suppression of IL 6 and TNF-*α*, as well as inhibition of COX-2 and iNOS activation via NF-kB pathway causing PGE2 and NO decrease. Based on preliminary reported data, the hydroxyl group of xanthorrhizol is essential for its pharmacological ability, in that acetylation of the hydroxyl group causes activity loss. However, in-depth future research is necessary to elucidate the structure-function correlation and molecular mode of action of xanthorrhizol.

In the most recent study, Kim et al. [54] reported the anti-inflammatory properties of standardized xanthorrhizol and *C. xanthorrhiza* extract in a high-fat diet- (HFD-) caused obese mouse. The report suggested the significant inhibition expression of inflammatory cytokines, such as C reactive protein (CRP), interleukin-1*β* (IL-1*β*), IL-6, and TNF-*α* in muscle (65.2–92.5%), liver (43.9–84.7%), and adipose tissue (27.8–82.7%) [54]. Repression of interleukin-1*β* (IL-1*β*) and nuclear factor kappa B (NF-kB) p65 by *C. xanthorrhiza* extract and xanthorrhizol has also been investigated on lipopolysaccharide- (LPS-) treated human gingival fibroblast-1 cells [91]. This result showed the potential of *C. xanthorrhiza* extract and xanthorrhizol to suppress LPS-activated oral inflammation. Thus, we believe that *C. xanthorrhiza* and xanthorrhizol have antiperiodontitis capability. In this research, Kim et al. [91] using xanthorrhizol at concentrations of 1 and 10 *μ*M (low and high, respectively). Hence, other concentrations between 1 and 10 *μ*g/ml *C. xanthorrhiza* or 1 and 10 *μ*M xanthorrhizol would have anti-inflammatory and antiosteoclastogenic activities. In addition, these data are reinforced by the ability of *C. xanthorrhiza* to inhibit the growth of periodontopathogens, such as *Streptococcus mutans* (Table 2). However, the stimulatory ability of *C. xanthorrhiza* and xanthorrhizol on osteoblastogenesis or the mechanism of bone development has not been illustrated yet. Hence, further studies on the osteoblastogenic effects and underlying molecular actions of these metabolites in living systems, such as animal models, are required.

Furthermore, some researchers used animal models to examine the anti-inflammatory potential of *C. xanthorrhiza* and its compounds. However, ethical processes increase the limitation of animal model-research based. Therefore, several in vitro anti-inflammatory approaches are available to elucidate the anti-inflammatory ability of *C. xanthorrhiza* and its isolated compounds. For example, flow cytometry is a fast and helpful instrument to discover the anti-inflammatory activity of some inflammatory markers together and there should be a useful anti-inflammatory investigation on *C. xanthorrhiza*.

### 8.4. Anticancer and Antitumor Properties

The first preliminary antitumor test of some compounds (*β*-atlantone, *α*-curcumene, *ar*-turmerone, and xanthorrhizol) from rhizome extract of *C. xanthorrhiza* was performed on sarcoma 180 ascites in rats [92]. Three compounds (*α*-curcumene, *ar*-turmerone, and xanthorrhizol) from this study showed significant antitumor activity evaluated by the packed cell volume method. The antimetastatic and antitumor potential of xanthorrhizol was further assessed in vivo using mice lung-metastasis specimens and a tumor cell development test [93]. Surprisingly, xanthorrhizol significantly suppressed the induction of tumor spots in the lung tissue and the development of the intra-abdominal mass of the tumor. This result is strengthened by the molecular analysis showing that xanthorrhizol could inhibit the expression of COX-2, phosphorylated extracellular signal-regulated kinase (ERK), and matrix metalloproteinase-9 (MMP-9) in metastatic mice [92]. Further assessment of isolated xanthorrhizol from *C. xanthorrhiza* against the proliferation of cancer cells was performed in combination with curcumin in MDA-MB-231 (human breast cancer cells) [22]. The experiment proved that the application of xanthorrhizol and curcumin exhibited synergistic growth inhibition on MDA-MB-231 cells *via* activation of apoptosis.

However, the above is not the first study reporting xanthorrhizol ability to induce cell apoptosis. Previous studies also showed that xanthorrhizol was able to activate apoptosis via induction of mitochondrial pathway (p53-dependent) in HeLa cervical cancer [94] and HepG2 liver cancer [94]. Within HeLa cervical cancer cells, xanthorrhizol upregulated p53 and Bax but had no effect on Bcl-2 (antiapoptotic protein). This upregulation of p53 and Bax protein production probably reactivates the sensitivity of cervical cancer cells toward apoptotic stimuli [94]. However, this result is in contrast with those reported by Handayani et al. [95] and Cheah et al. [22], where upregulation of p53 did not affect Bax expression but lowered the Bcl-2 level in HepG2 liver cancer cells and MCF-7 breast cancer. From these findings, xanthorrhizol may have apoptosis induction activity via p53-regulated mitochondrial pathway in particular cancer cells with diverse regulation on the expression of Bax/Bcl-2 [86].

### 8.5. Antidiabetic Properties

The antimetabolic disorder effect of *C. xanthorrhiza* extract, such as antidiabetic, has been evaluated using both insulin-dependent [53] and no-insulin-dependent diabetic models [54]. Yasni et al. [53] reported the antidiabetic properties of the extract of *C. xanthorrhiza* in the streptozotocin-activated diabetic mouse. The report suggested that the extract of *C. xanthorrhiza* extremely lowered the level of serum glucose and triglyceride as compared to cellulose and other tested herbals. *C. xanthorrhiza* was also found to reduce the ratio of arachidonate to linoleate in the liver phospholipids [53]. In another study using high-fat diet- (HFD-) induced obese rats, Kim et al. [54] found that both xanthorrhizol and *C. xanthorrhiza* extracts could exceptionally suppress postprandial blood glucose content in HFD-induced obese mouse. Moreover, xanthorrhizol and *C. xanthorrhiza* extracts are also able to reduce triglyceride (TG), glucose, free fatty acid (FFA), and insulin composition in serum [54]. According to this study, xanthorrhizol and *C. xanthorrhiza* extracts may suppress and cure noninsulin-dependent diabetes, which is mostly caused by obesity-activated resistance of insulin.

### 8.6. Skin Care

The study of anti-skin aging properties of xanthorrhizol was performed by evaluating its effect on the expression of MMP-1 and type-1 procollagen in UV-irradiated human skin fibroblasts [96]. Matrix metalloproteinases (MMPs) are one responsible factor mediating the UV-induced skin aging that is upregulated by UV irradiation. Meanwhile, type-1 collagen is the main builder of the skin dermis, and the degradation of this structural protein could lead to skin aging problems. Xanthorrhizol isolated from *C. xanthorrhiza* was proven to be efficacious for decreasing the expression level of MMP-1 and increasing type-1 procollagen expression [96]. Xanthorrhizol's ability to suppress the MMP-1 expression and increase type-1 procollagen expression is even better than the effect of a natural antiaging agent known as epigallocatechin 3-O-gallate (EGCG) [95].

In addition, skin-caring effects of *C. xanthorrhiza* were also evaluated using its flower bract extract. Batubara et al. [47] reported that methanol extract of *C. xanthorrhiza* flower bract could suppress the growth of acne-causing microbe *Propionibacterium acnes* and its ethyl acetate flower bract extract could suppress tyrosinase and lipase activities. *α*-curcumene and xanthorrhizol of *C. xanthorrhiza* flower bract extract are two main compounds responsible for lipase activity inhibition and *Propionibacterium acnes* growth suppression, respectively [47]. This study shows that *C. xanthorrhiza* flower bract extract is a potent natural skincare and whitening agent. However, this study did not assess dose-response effects. Moreover, since the study of flower bract extract of *C. xanthorrhiza* is still limited, a follow-up study is required to reveal other active molecules within the flower bract extract that is responsible for the skin-caring activity as well as its possible structure-function relationship.

### 8.7. Hepatoprotective Activity

Oral application of *C. xanthorrhiza* in mice and rats has been shown to have hepatoprotective effects from a number of hepatotoxic causes, such as acetaminophen, galactosamine, and carbon tetrachloride, as indicated by extreme alleviation of serum transaminases [12, 97]. This hepatoprotective activity is regarded due to the presence of its antioxidant compounds, as well as its capability to reduce the activation of proinflammatory cytokines. Kim et al. [15, 56] found that oral feeding of 200 mg xanthorrhizol weakened cisplatin-induced hepatotoxicity and nephrotoxicity in mice (cisplatin is frequently applied in chemotherapy). Its likely mode of action is by attenuation of c-Jun N-terminal kinases (JNKs) phosphorylation. The above effect shows that *C. xanthorrhiza* has the potential to safeguard the liver against various hepatotoxins as also reported by many of its traditional uses. This hepatoprotective activity was further supported by other reports showing that *C. xanthorrhiza* could reduce liver triglyceride level and serum triglyceride content [18, 55].

## 9. Safety Evaluation (Cytotoxicity)

Oral administration of up to 2 g/kg body weight of aqueous extract of *C. xanthorrhiza* showed no indication of toxicity in mice or rats [97]. A previous study also showed that no mortality was found following a single oral application of pure xanthorrhizol to mice at 500 mg/kg [98]. In the most recent report, the toxicity level of ethanol extract of *C. xanthorrhiza* has been evaluated orally on the hematological and male reproduction system of mice [99]. The obtained results suggested that oral application of *C. xanthorrhiza* had no significant effect on hematological and spermatogenic changes. Hence, *C. xanthorrhiza* did not give a toxic effect on the hematological characteristics and reproductive system of male mice [99]. This result is in contrast to *C. zedoaria* Roscoe. causing a notable decrease in RBC, Hb level, and spermatozoa quality [99].

Although in vivo tests of aqueous extract of *C. xanthorrhiza* (at dose 2 g/kg) and pure xanthorrhizol (500 mg/kg dosage) are shown to be nontoxic to mice, prior to clinical trials, we propose that in vivo mice pharmacokinetic and pharmacodynamic tests must be performed in depth. The logical reason is that in the in vivo systems all the processes of absorption, distribution, metabolism, and elimination of a compound are much more complex than the in vitro systems [100]. Pharmacokinetic evaluation is needed to increase the efficacy of the extract or compound being tested, as well as to lessen the level of toxicity. In a complex living system, the connection between the concentration of the drug at the action area and its pharmacological reaction could be predicted via pharmacodynamics methods [100]. In addition, scientific data on the mutagenic potential of *C. xanthorrhiza,* genotoxicity, carcinogenicity, and even reproductive toxicity of *C. xanthorrhiza* is still limited. Therefore, future studies of efficacy and safety dosage of *C. xanthorrhiza* extract and its active compounds in targeted therapeutic areas are compulsory. However, it is not recommended to use *C. xanthorrhiza* or preparations thereof during breastfeeding since curcumin and/or active compounds are transferred to sucklings via lactation.

## 10. Conclusion and Perspective

The present review summarizes comprehensive information about botany and distribution, traditional medication uses, production and conservation status, phytochemical attributes, and pharmacological properties of *C. xanthorrhiza* Roxb. The preliminary pharmacodynamic data shows that *C. xanthorrhiza* may strengthen bile secretion sometimes following oral administration [53, 101]. The main active components to trigger this action have been shown to be curcumin and camphor [53, 101]. This could be related to the traditional application of *C. xanthorrhiza* in digestive disorder due to bile secretion deficiency, such as flatulence, slow digestion, and feelings of fullness. However, the quantity of metabolite required to produce this effect seems to be much greater than the traditional dose. Hence, the reference to the effect on bile secretion should be omitted from the indication. Based on this consideration, there is no need for contraindication for biliary obstruction. However, caution is taken in case of biliary obstruction. Nevertheless, more comprehensive pharmacokinetic and pharmacodynamics evaluation should be conducted before clinical test.

Furthermore, some issues regarding this *C. xanthorrhiza* still remain unclarified. Firstly, *C. xanthorrhiza* is commonly combined with other jamu plants (such as *Zingiber officinale* and *Curcuma longa*) in traditional therapies. The synergetic effects and underlying mechanisms between *C. xanthorrhiza* and other *jamu* plants should be studied in detail. Secondly, though rhizome is traditionally used as *jamu* ingredient, some scientists investigated the active component of other parts (such as flower bract) and demonstrated their pharmacological activities. Therefore, it is essential to compare the secondary metabolites and corresponding biological activities among various organs of this plant. Thirdly, reported pharmacological studies mostly focused on a limited number of metabolites (mainly xanthorrhizol and curcumin). Therefore, we need to answer if these explored chemicals can achieve the equivalent effect of *C. xanthorrhiza*, or, if not, to what extent. Otherwise, more phytochemicals should be elucidated using bioactivity-guided isolation methods. Fourthly, the molecular mechanisms underlying the biological activities of terpenoids and curcuminoids of *C. xanthorrhiza* remain inadequate. Thus, we believe that further investigations should focus on the absorption, metabolism, distribution, and excretion pathways of those main major groups of chemicals within *C. xanthorrhiza*. We believe that if the above questions are successfully answered in depth and identify more medical potential of *C. xanthorrhiza,* we can take more benefit from it for the sake of human health.

In conclusion, though many reports already exist on the local uses, isolated active compounds, and pharmacological activities of *C. xanthorrhiza*, some important research space still needs to be addressed such as comprehensive toxicity tests, optimized biotechnological production, and extensive clinical study to support its sustainable commercial application. Nevertheless, this review could be used as a basis for further research and commercialization of this plant and lead to the validation of unconfirmed ethnomedicinal claims.

## Figures and Tables

**Table 1:** Ethnomedicinal uses of *C. xanthorrhiza* among Indonesian local communities.

NO	Local community	Traditional uses	Ethnomedicinal use categories [28]	References
1	Aceh	Blood booster and malaria medication	Cardiovascular diseases, Parasites	[29]
2	Bali	Stomach disorders medication	Gastrointestinal problems	[29]
3	Banjarbaru	Blood neutralizing, respiratory disorders, muscular disorders, head disorders, and colds	Metabolic syndromes, Cardiovascular diseases, Respiratory complaints, Skeletomuscular system	[30]
4	Enggano, North Bengkulu	Postpartum medicine	Gynaecology	[31]
5	Kaili Ledo, Sigi	Treating back pain	Skeletomuscular system	[32]
6	Madura	Vaginal discharge	Gynaecology	[29]
7	Sakai, Bengkalis	Appetite booster	Gynaecology	[29]
8	Sinjai, South Celebes	Stomach disorders medication	Gastrointestinal problems	[33]
9	Sukolilo, Surabaya	Maintain health, eliminate fatigue, and maintain body fitness	Metabolic syndromes	[34]
10	Sumowono, Semarang	Cleaning the digestive tract, increase appetite, treatment after typhus, post-liver disease, eliminate fatigue	Gastrointestinal problems, metabolic syndromes	[35]
11	Surakarta	Wedding potion	Gynaecology	[36]
12	Urug, Sukajaya, Bogor	Postdelivery concoctions	Gynaecology	[37]
14	West Java	Treating jaundice, flatulence, urinary stones	Metabolic syndromes, gastrointestinal problems, urology	[29, 38]

**Table 2:** Antibacterial, antifungal, and anti-insecticidal activities of *C. xanthorrhiza rhizome* extract.

No	Potential Disease category	Extract type	Dose concentration	Tested species	Effect	Reference
*Antibacterial*						
1	Dental plaque	Pure xanthorrhizol	5 *µ*mol/L	*Streptococcus mutans* ATCC 25175	Complete inhibition of biofilm formation	[40]
		70% ethanol rhizome extract	MIC: 0.1% (*w*/*v*)	*Streptococcus mutans type F* (*MUI*)	Bacterial growth inhibition	[41]
2	Food-borne illness	Pure xanthorrhizol	MIC: 8 *µ*g/ml; MBC: 16 *µ*g/ml	*Bacillus cereus ATCC 21772*	Treatment at 4x MIC reduced viable cells by at least 6 to 8 log in 4 h	[42]
			MIC: 16 *µ*g/ml; MBC: 32 *µ*g/ml	*Clostridium perfringens ATCC 3624*	Treatment at 4x MIC reduced viable cells by at least 6 to 8 log in 4 h	[42]
			MIC: 8 *µ*g/ml; MBC: 16 *µ*g/ml	*Listeria monocytogenes ATCC 15313*	Treatment at 4x MIC reduced viable cells by at least 6 to 8 log in 4 h	[42]
			MIC: 8 *µ*g/ml; MBC: 16 *µ*g/ml	*Staphylococcus aureus KCCM 11764*	Treatment at 4x MIC reduced viable cells by at least 6 to 8 log in 4 h	[42]
			MIC: 16 *µ*g/ml; MBC: 16 *µ*g/ml	*Salmonella typhimurium KCCM 11862*	Treatment at 4x MIC reduced viable cells by at least 6 to 8 log in 4 h	[42]
			MIC: 8 *µ*g/ml; MBC: 16 *µ*g/ml	*Vibrio parahaemolyticus ATCC 17802*	Treatment at 4x MIC reduced viable cells by at least 6 to 8 log in 4 h	[42]
		70% ethanol rhizome extract	MIC: 2.0% (*w*/*v*)	*Bacillus cereus ATCC 11778*	Bacterial growth inhibition	[41]
3	Skin diseases	70% ethanol rhizome extract	MIC: 0.1% (*w*/*v*)	*Staphylococcus aureus ATCC 25923*	Bacterial growth inhibition	[41]
4	Infectious diseases	96% ethanol rhizome extract	100% extract concentration	*Staphylococcus epidermidis ATCC 12228*	Showed average inhibition zone diameter of 9.20 mm	[43]
5	Pneumonia	100% methanol rhizome extract	MIC:1.25 mg/ml; MBC: 2.5 mg/ml	*Klebsiella pneumonia ATCC 13773*	The bacteria completely killed at 4x MIC for 15 min	[44]
6	Tuberculosis	96% ethanol rhizome extract	MIC: 1600 *µ*g/ml; MBC: 3200 *µ*g/ml	*Mycobacterium tuberculosis* H37Rv	Bacterial growth inhibition	[44]
7	Pulp necrosis	Essential oil extract	25%–100% extract concentration	*Enterococcus faecalis*	Inhibition zone: 8–11 mm (medium level antibacterial)	[45]
				*Fusobacterium nucleatum*	Inhibition zone: 8–11 mm (medium level antibacterial)	[45]
8	Diarrhea	96% ethanol rhizome extract + nanoparticles	500 *µ*g/ml	*Escherichia coli*	Bacterial growth inhibition	[46]
9	Typhus	96% ethanol rhizome extract + nanoparticles	500 *µ*g/ml	*Salmonella typhi*	Bacterial growth inhibition	[46]
		96% ethanol rhizome extract + nanoparticles	500 *µ*g/ml	*Bacillus subtilis*	Bacterial growth inhibition	[46]
10	Acne	Essential oil of flower bract	MIC: 1 mg/ml; MBC: 3 mg/ml	*Propionibacterium acnes*	Bacterial growth inhibition	[47]

*Antifungal*						
11	Nosocomial infections	Pure xanthorrhizol	8 *µ*g/mL	*Candida albicans*	Inhibition of biofilm formation and conidial germination	[48]
12	Hospital-acquired infection	Pure xanthorrhizol	MIC: 1.0–10 mg/l; MFC: 15 mg/ml	*Candida glabrata*	Able to kill the Candida strains	[40]
			MIC: 2.0–8.0 mg/l; MFC: 12.5 mg/ml	*Candida guilliermondii*		
			MIC: 2.5–7.5 mg/l; MFC: 10 mg/ml	*Candida krusei*		
			MIC: 2.5–25 mg/l; MFC: 30 mg/ml	*Candida parapsilosis*		
			MIC: 2.0–8.0 mg/l; MFC: 10 mg/ml	*Candida tropicalis*		
13	Filamentous fungal infections	Pure xanthorrhizol	MIC: 2.0 *µ*g/ml; MFC: 4.0 *µ*g/ml	*Aspergillus flavus*	Inhibition the conidial germination	[49]
			MIC: 2.0 *µ*g/ml; MFC: 4.0 *µ*g/ml	*Aspergillus fumigatus*		
			MIC: 2.0 *µ*g/ml; MFC: 4.0 *µ*g/ml	*Aspergillus niger*		
			MIC: 4.0 *µ*g/ml; MFC: 8.0 *µ*g/ml	*Fusarium oxysporum*		
			MIC: 1.0 *µ*g/ml; MFC: 2.0 *µ*g/ml	*Rhizopus oryzae*		
			MIC: 1.0 *µ*g/ml; MFC: 2.0 *µ*g/ml	*Trichophyton mentagrophytes*		
14	Skin and nail infections	Acetone extract and chloroform fractions	MIC:12.5–25.0 *μ*g/mL	*Epidermophyton* sp.	Significant growth inhibition	[50]
15				*Trichophyton rubrum*		
16	Penicilliosis	Acetone extract and chloroform fractions	MIC:12.5–25.0 *μ*g/mL	*Penicillium* sp.	Significant growth inhibition	[50]

*Anti-insecticidal*						
17	Agriculture pest	Pure xanthorrhizol	LD50 = 6.92–8.13 *μ*mol/kg fr.wt	*Spodoptera littoralis*	Significant mortality of the larvae	[51]
